# From Nature to Medicine: Overcoming the Limitations of Herbal Therapeutics for Pancreatic Cancer Treatment Based on Nanotechnology

**DOI:** 10.3390/nano16191256

**Published:** 2026-10-04

**Authors:** Ghazala Muteeb, Youssef Basem, Amira A. Kotb, Naiera T. Magdy, Ghannimah Yousef Alghuwainem, Mohammad Aatif, Mohd Farhan, Doaa S. R. Khafaga

**Affiliations:** 1 Department of Nursing, College of Applied Medical Sciences, King Faisal University, Al-Ahsa 31982, Saudi Arabia; gyalghuwainem@kfu.edu.sa; 2 Medical and Pharmaceutical Industrial Biotechnology Department, College of Biotechnology, Misr University for Science and Technology (MUST), 6th of October City 12566, Egypt; 200025117@must.edu.eg; 3 Department of Chemistry and Biochemistry, Faculty of Science, Cairo University, Giza 12613, Egypt; 202228238@std.sci.cu.edu.eg; 4 Faculty of Biotechnology, German International University (GIU), New Administration Capital 11835, Egypt; naieratamer21@gmail.com; 5 Department of Public Health, College of Applied Medical Sciences, King Faisal University, Al-Ahsa 31982, Saudi Arabia; maahmad@kfu.edu.sa; 6 Department of Chemistry, College of Science, King Faisal University, Al-Ahsa 31982, Saudi Arabia; mfarhan@kfu.edu.sa; 7 Department of Basic Medical Sciences, Faculty of Medicine, Galala University, New Galala City 43511, Egypt

**Keywords:** pancreatic cancer, nanomedicine, green synthesis, herbal phytochemicals, pharmacokinetics, biopolymer-assisted nanoparticles, targeted drug delivery, translational oncology

## Abstract

Pancreatic ductal adenocarcinoma (PDAC) remains one of the most lethal malignancies worldwide due to late-stage diagnosis, aggressive progression, and limited therapeutic responsiveness. Although herbal-derived bioactive compounds have demonstrated promising anticancer potential, their clinical application is often restricted by poor aqueous solubility, low bioavailability, rapid metabolic degradation, and insufficient tumor targeting. Nanomedicine has emerged as a promising platform to overcome these limitations through enhanced drug delivery, improved pharmacokinetics, and controlled release capabilities. In recent years, the integration of green nanotechnology with herbal therapeutics has gained increasing attention as a sustainable and biocompatible strategy for PDAC management. Green synthesis approaches utilizing plant extracts, microbial systems, and biopolymers provide environmentally friendly alternatives to conventional physicochemical nanoparticle (NP) fabrication methods while simultaneously enhancing biological functionality. This review aims to explore green nanotechnology-based strategies for improving the therapeutic potential of herbal-derived bioactive compounds in PDAC, with particular emphasis on nanoformulation approaches that enhance solubility, bioavailability, tumor targeting, and anticancer efficacy. In addition, the review discusses the advantages of green synthesis approaches, NP characterization strategies, translational barriers, biosafety considerations, and current clinical challenges. Overall, this review highlights the growing potential of green nanomedicine to improve herbal-based therapeutic strategies against PDAC while emphasizing the key obstacles that must be addressed to facilitate successful clinical translation.

## 1. Introduction

**Figure 1:**
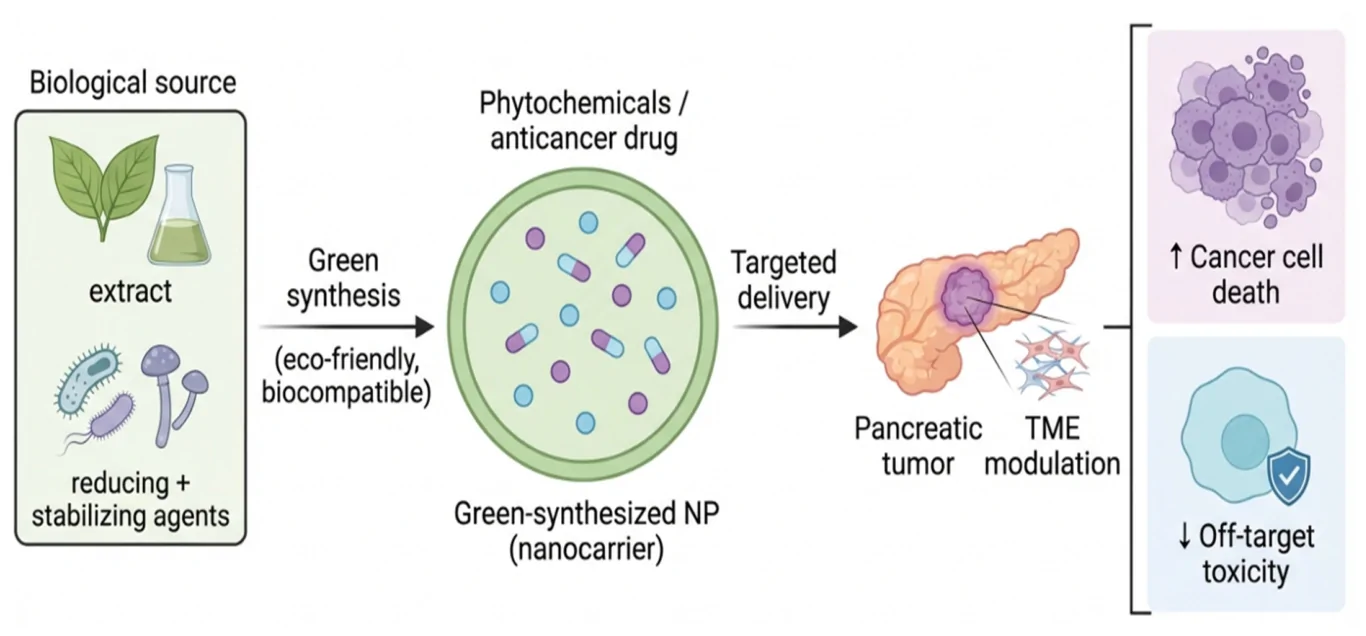
Schematic illustration of the green synthesis of NPs and their multifaceted role in PC therapy. The diagram highlights the utilization of biological extracts as eco-friendly reducing and stabilizing agents to fabricate biocompatible nanocarriers. These NPs facilitate targeted drug delivery to the pancreatic tumor site, modulate the tumor microenvironment (TME), and result in enhanced pro-apoptotic activity while significantly reducing systemic off-target toxicity.

Pancreatic ductal adenocarcinoma represents one of the most aggressive and treatment-resistant malignancies, characterized by late diagnosis, extensive desmoplastic stroma, limited drug penetration, and poor clinical outcomes. Despite advances in chemotherapy and targeted approaches, therapeutic efficacy remains restricted by intrinsic resistance mechanisms and systemic toxicity, highlighting the urgent need for innovative treatment strategies [1]. PDAC represents a major therapeutic challenge due to its aggressive biological behavior, late diagnosis, extensive desmoplastic stroma, poor vascular perfusion, and profound resistance to conventional therapies. These characteristics collectively limit drug penetration and reduce the effectiveness of currently available treatment strategies, highlighting the need for innovative therapeutic approaches. Special types of green-synthesised NPs are nanomaterials that can be made in an eco-friendly manner by using biomaterials, such as fungi, bacteria, or plant extracts, as stabiliszing and reducing agents, as shown in Figure 1. This environmentally friendly method uses less harmful chemicals and has a smaller environmental impact, frequently producing biocompatible NPs for biomedical applications. Green synthesis fully utilizes the qualities of natural sources, offering a viable, affordable, sustainable, and scalable alternative to traditional chemical approaches [2].

Cancer is fundamentally a genetic disease driven by the accumulation of genetic and epigenetic alterations that affect key cellular processes, including genome stability, tumour suppression, and oncogenic signalling. However, cancer development and progression are also strongly influenced by extrinsic factors, including the tumor microenvironment, stromal components, and immune regulation [3]. Only when tumour cells are able to disrupt immune control can they proliferate and eventually kill their host [4].

Today’s medical professionals are looking to traditional medicine for ideas to improve current treatment methods by integrating technology with herbal knowledge [5]. Phytomedicine, another name for herbal medicine, uses bioactive substances derived from plants and herbs to improve general health and well-being [6]. Natural products have served as important sources of potent anticancer agents, including several clinically approved drugs. These include the vinca alkaloids vincristine, vinblastine, vindesine, and vinorelbine, as well as the taxanes paclitaxel (PTX), docetaxel, podophyllotoxin and its derivatives, etoposide, teniposide, and several other plant-based drugs that have been approved for use in cancer treatment by the US Food and Drug Administration (US FDA) [7]. However, the frequency of tumor cells that exhibit resistance to standard therapeutic approaches, including chemotherapy and radiotherapy, while surgical limitations may arise from tumor location, invasion, or disease stage, radiation, and surgery, as well as the fact that these treatments result in a number of negative reactions and unsatisfactory treatment outcomes, highlights the urgent need for the advancement and development of antitumor therapeutic research that has the potential to target tumors and effectively kill cancer cells with minimal side effects [8].

Due to their exceptional safety and effectiveness, cancer nanomedicines, also referred to as “magic bullets “, are now often used in the treatment of cancer. Compared with traditional medication administration techniques, using NPs in cancer treatment has significantly improved drug delivery to the target site. It greatly improves the efficacy and safety of the most widely used anticancer medications. Prolonged half-life, delayed release, efficient targeting, and decreased toxicity are major advantages of these delivery methods and nanomedicine [9]. Using these methods for several types of cancers, especially pancreatic cancer (PC), which has an average five-year survival rate of just 9%, PC ranks as the seventh most common cause of cancer-related mortality globally. According to GLOBOCAN 2018 estimates, PC accounted for approximately 458,918 new cases and 432,242 deaths worldwide in 2018. These incidence and mortality estimates represent separate population-based measures and do not indicate that all deaths occurred among patients newly diagnosed within the same year [10]. PCs comprise several histological subtypes, with PDAC representing the predominant form, accounting for approximately 85% of cases and arising from the exocrine pancreas. Pancreatic neuroendocrine tumors represent a less common endocrine subtype, accounting for fewer than 5% of pancreatic malignancies. Other rare pancreatic tumor types, including acinar cell carcinoma and additional uncommon neoplasms, account for the remaining cases [4]. The primary objective of this study is to explore the potential of green-synthesized NPs as effective and sustainable therapeutic agents for PC. Specifically, the research aims to evaluate their anticancer efficacy, biocompatibility, and mechanism of action, while emphasizing their roles in targeted drug delivery and modulation of the tumour microenvironment. This work seeks to bridge nanotechnology, phytochemistry, and oncology, providing a foundation for developing eco-friendly, nanomedicine-based approaches to treat highly lethal malignancies such as PC. This review focuses on integrating herbal-derived bioactive compounds with green nanotechnology-based delivery systems for PDAC management. Rather than discussing nanomedicine as an independent therapeutic field, nanoplatforms are examined as enabling tools designed to overcome the pharmacological limitations of herbal agents, including poor aqueous solubility, limited bioavailability, rapid degradation, and inadequate tumor accumulation. Therefore, this review discusses integrating herbal-derived bioactive compounds with green nanotechnology-based platforms for PDAC treatment. The review focuses on (i) the therapeutic barriers associated with PDAC, (ii) the pharmaceutical limitations of herbal compounds, (iii) green synthesis approaches and nanocarrier systems, and (iv) recent advances and challenges related to the clinical translation of herbal nanomedicine.

## 2. Methodology

This narrative review was conducted through a comprehensive literature search of peer-reviewed publications between 2010 and 2025 using multiple academic databases, including Scopus, PubMed, Web of Science, and ScienceDirect. The retrieved literature was critically evaluated and thematically organized to provide an overview of recent advances, challenges, and future perspectives in green nanomedicine and herbal-based therapeutic strategies for pancreatic cancer using multiple academic databases, including Scopus, PubMed, Web of Science, and ScienceDirect. The search strategy employed Boolean combinations of the keywords “green nanomedicine,” “green synthesis,” “phytochemical nanoparticles,” “biogenic nanoparticles,” “nanocarriers,” “drug delivery,” and “pancreatic cancer.” Only English-language articles and reviews focusing on the development, characterization, and therapeutic evaluation of green or biogenic nanomedicines for pancreatic cancer were included. Exclusion criteria comprised non-cancer applications, studies unrelated to environmentally friendly synthesis approaches, and non-original publications such as editorials or conference abstracts. Preference was given to peer-reviewed studies emphasizing mechanistic insights, in vitro and in vivo validation, and translational relevance. Retrieved articles were thematically categorized into: (1) green synthesis and characterization of nanocarriers, (2) biological activities and anticancer mechanisms, (3) pharmacokinetics and toxicity profiles, and (4) preclinical or clinical therapeutic outcomes.

## 3. Herbal Limitations for Pancreatic Cancer Treatment

Despite their relatively high safety profile, many plant-derived natural compounds suffer from poor aqueous solubility and limited absorption, which may restrict their bioavailability and therapeutic application [11]. Contemporary nanodrug delivery techniques can help overcome these drawbacks. Herb–Drug Interactions (HDIs) have two different impacts [12]. Herbs have the potential to reduce the effectiveness, selectivity, and even toxicity of medicinal medications. For instance, when taken with Warfarin, Ginkgo biloba L. and Salvia miltiorrhiza Bunge cause adverse reactions (ADR), including bleeding [13]. However, when herbs are used properly, they can improve the bioavailability, efficacy, and ADR of medicinal medications. Numerous investigations have demonstrated that *Curcuma longa* L. and doxorubicin (DOX) act in concert to inhibit the growth, migration, and invasion of cancer cells while reducing DOX-induced cardiotoxicity and nephrotoxicity [14,15,16,17,18]. As summarized in Table 1, herbal-derived compounds such as polyphenols and alkaloids exhibit significant therapeutic potential due to their antioxidant, anticancer, and cytotoxic properties. However, their clinical application is often limited by drawbacks such as poor solubility, bioavailability, and systemic toxicity.

## 4. Nanomedicine in Pancreatic Ductal Adenocarcinoma: Overcoming Stromal and Vascular Barriers to Drug Delivery

Pancreatic ductal adenocarcinoma remains one of the deadliest cancers, with its dismal prognosis largely attributable to its distinct pathophysiological characteristics. A central barrier is its deficient vascular network; the tumor is supplied by irregular, scarce microvessels that generate elevated interstitial fluid pressure, collectively restricting effective perfusion and limiting the delivery of chemotherapeutic agents [26]. Adding to this challenge is the dense desmoplastic stroma, a fibrotic tissue network largely composed of cancer-associated fibroblasts (CAFs), pancreatic stellate cells, extracellular matrix components, and immunosuppressive cells. This stroma, which can account for as much as 90% of the tumor mass, forms a substantial physical barrier that severely restricts drug delivery [27]. Moreover, drug resistance in PDAC arises from multiple factors. On the intrinsic side, genetic mutations such as those in KRAS and TP53, epigenetic modifications, upregulation of efflux transporters, and persistent cancer stem cell subsets drive resistance. Externally, resistance is reinforced by the desmoplastic stroma, low-oxygen conditions, and a microenvironment that suppresses immune activity [28]. The convergence of these barriers underscores the urgent need for nanomedicine strategies that can navigate the complex tumor microenvironment of PDAC.

**Figure 2:**
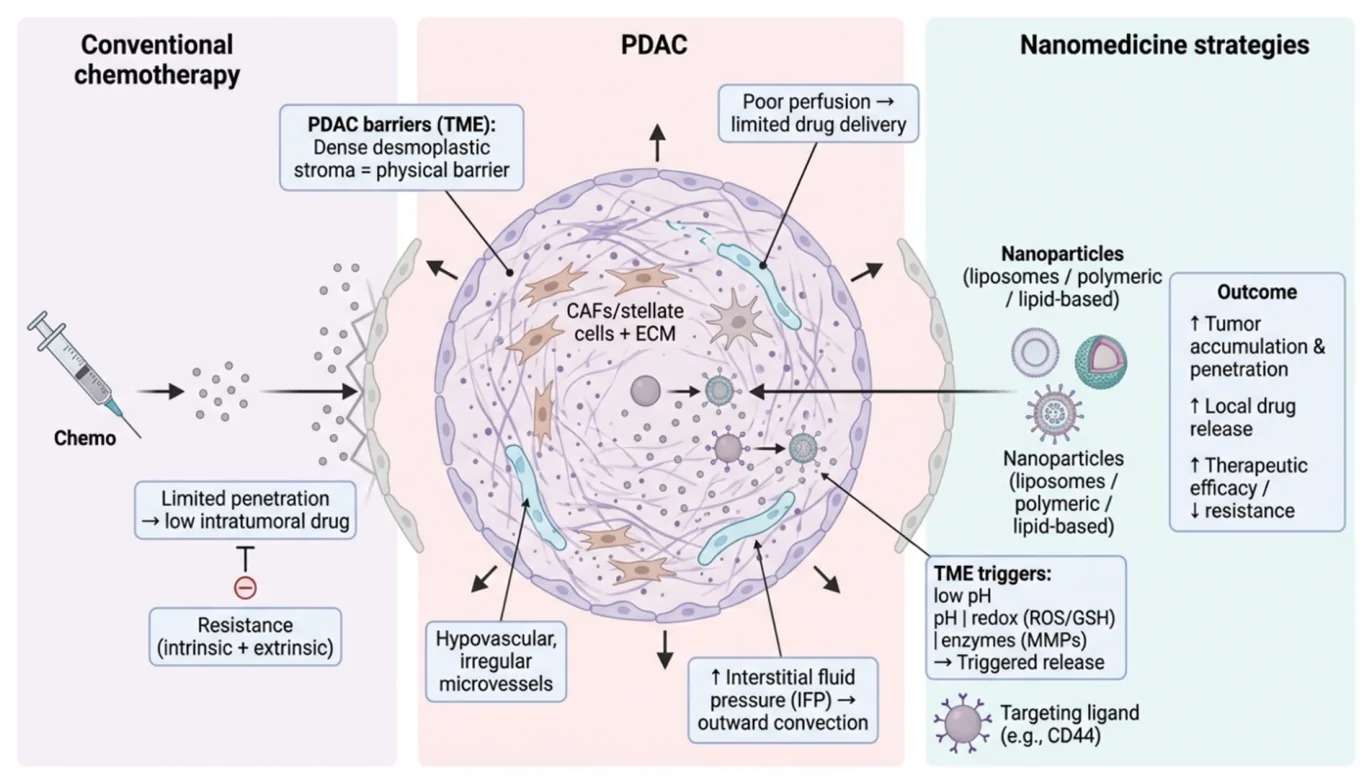
Mechanistic overview of nanomedicine-mediated TME remodeling in PDAC.

The complex biological barriers of PDAC represent major obstacles to effective therapeutic delivery. Understanding these barriers is essential for designing advanced delivery strategies, including nanotechnology-based platforms, which will be discussed in subsequent sections [29]. In oncology, nanomedicine offers unique advantages including enhanced permeability and retention (EPR) effects that allow preferential accumulation of NPs within tumor tissues. These properties make nanotechnology-based systems promising tools for overcoming biological barriers and improving therapeutic outcomes [30]. Integrating nanomedicine into PDAC management has gained attention for its potential to overcome stromal and vascular barriers that limit conventional chemotherapy efficacy, as shown in Figure 2. NPs can improve intratumoral drug penetration, reduce off-target effects, and achieve sustained drug release, thereby increasing therapeutic efficacy and patient survival rates [31]. Nanomedicine provides a diverse set of nanocarrier systems aimed at improving therapeutic delivery across poorly vascularized and stroma-rich tumors. Clinically approved examples include liposomal irinotecan (nal-IRI, Onivyde^®^), which achieves extended circulation times and enhanced tumor deposition through the EPR effect, and albumin-bound paclitaxel (nab-PTX, Abraxane^®^), which increases drug solubility and facilitates uptake via receptor-mediated mechanisms [32].

Polymeric NPs offer substantial drug-loading capacity and controllable release profiles while maintaining strong biocompatibility. They have been investigated as carriers for chemotherapeutic drugs, nucleic acid therapeutics, and agents that modulate the immune response in PDAC models [33]. Dendrimers are extensively branched macromolecules that enable fine-tuned surface modification and the attachment of multiple ligands, thereby supporting targeted drug delivery. However, their application in PDAC remains relatively limited, with studies still emerging. Combinations such as polymer–lipid hybrids or inorganic–organic composites provide improved stability, multifunctional design, and controlled drug release. For instance, lipid–polymer hybrid NPs have demonstrated prolonged circulation times and effective protection of therapeutic cargo [34]. Solid lipid nanoparticles (SLNs) composed of biocompatible lipids offer advantages such as lower toxicity, prolonged circulation time, greater solubility, and enhanced drug bioavailability. In PDAC models, recent investigations highlight the promise of these systems in overcoming stromal barriers and facilitating efficient delivery of hydrophobic therapeutics [35]. In addition, recent innovations include olive oil–derived liquid nanocapsules conjugated with CD44 antibodies, which have demonstrated up to a fourfold increase in paclitaxel uptake by PC stem cells, both in vitro and in xenograft models [36].

Stimuli-responsive NPs are engineered to react to tumor-specific signals, enabling controlled and site-specific drug release. pH-sensitive platform systems exploit the acidic environment of the tumor extracellular space and endolysosomal compartments to initiate drug release. Certain polymer–lipid hybrids and micellar carriers employ pH shifts as a trigger for regulated delivery. The high levels of reactive oxygen species (ROS) and glutathione commonly present in tumor cells can cleave disulfide linkages within NPs, thereby liberating their therapeutic payloads. NPs sensitive to redox conditions are increasingly recognized as a promising smart delivery strategy [37]. Certain enzymes, including matrix metalloproteinases (MMPs) and proteases linked to the fibrotic stroma, can cleave tailored linkers within nanocarriers, enabling targeted drug release within the hostile microenvironment of PDAC [28]. New generations of “smart” nanocarriers integrate multiple triggers such as pH changes, redox conditions, and enzymatic activity to enable precise spatiotemporal drug release. This approach improves tumor accumulation and cellular uptake while reducing off-target toxicity [28]. A recent review on responsive nanomaterial-based delivery strategies emphasizes their potential to bypass PDAC’s limited vascularization and dense stromal barrier by enabling stimulus-triggered drug release deep inside the tumor tissue [38].

These biological and physicochemical barriers highlight the need for advanced delivery systems capable of improving therapeutic accumulation and efficacy in PDAC. The following sections discuss nanotechnology-based platforms designed to address these challenges.

## 5. Types of Nanoparticles Used for Pancreatic Cancer Treatment

Although various nanocarrier platforms have been developed for PDAC therapy, this review focuses on those that can encapsulate, protect, and enhance the therapeutic performance of herbal-derived molecules. The NP platforms discussed in this section are selected based on their relevance to herbal compound delivery and green nanotechnology. Conventional non-herbal nanoformulations are included only to provide mechanistic context and comparison. NPs are increasingly recognized as transformative carriers in PC treatment due to their ability to enhance targeted delivery and overcome key therapeutic barriers [26]. They exploit their small size and the EPR effect to preferentially accumulate within tumor tissue, thereby improving local drug concentrations while reducing systemic side effects [26]. Diverse NP platforms, including liposomes, polymeric nanoparticles, micelles, and inorganic nanoparticles, are under active development for PDAC, each offering unique properties for encapsulating chemotherapeutics, biological drugs, or combination cargos [39]. By enabling controlled release, improving circulation time, and facilitating co-delivery strategies, these nanocarriers address the critical challenge of delivering therapeutic payloads to poorly perfused, dense pancreatic tumors [40]. Beyond drug delivery, NPs also facilitate modulation of the immune microenvironment and stromal architecture in PDAC, enabling immunotherapeutic strategies, disruption of desmoplasia, and enhanced penetration into hypervascular tumor regions [41]. Altogether, NP-enabled therapeutics offer a multifaceted toolset with the potential to transform PC treatment by addressing both delivery limitations and biological resistance mechanisms.

The selection of nanocarrier platforms for PDAC therapy is primarily driven by the unique biological barriers imposed by this malignancy, particularly the dense desmoplastic stroma, poor vascular perfusion, elevated interstitial pressure, and profound therapeutic resistance. Therefore, NP classes discussed in this review were selected based on their ability to address specific PDAC-associated challenges through distinct physicochemical properties. Lipid-based and polymeric NPs provide improved solubility, prolonged circulation, and controlled release of hydrophobic herbal compounds, whereas metallic NPs offer tunable surface chemistry, photothermal capabilities, and facile functionalization with targeting ligands. Carbon-based nanomaterials provide high surface area and multifunctional platforms for drug loading and photothermal applications, while stimuli-responsive systems, such as ADAM9-responsive mesoporous silica NPs, enable tumor-specific activation and localized drug release within the PDAC microenvironment. Collectively, these platforms were selected because their colloidal properties, surface engineering potential, and biological responsiveness provide complementary strategies to overcome stromal barriers and enhance therapeutic delivery in pancreatic cancer [40].

### 5.1. Metallic NPs

#### 5.1.1. Gold NPs

In the context of green nanomedicine, gold nanoparticles (AuNPs) synthesized using plant-derived reducing agents have attracted attention due to their ability to combine the intrinsic bioactivities of phytochemicals with the delivery advantages of nanoscale systems [42]. AuNPs also act as multifunctional theragnostic platforms by combining photothermal therapy (PTT) with drug delivery and imaging modalities (photoacoustic, CT), and by serving as carriers for small-molecule drugs or biologics to enable chemo-photothermal combination regimens [43]. Penetration studies demonstrate that two- and three-layered AuNPs achieve significantly greater uptake in pancreatic tumor cell cultures than conventional PEGylated nano shells, with their superior transport properties enabling more effective traversal of tumor-associated vascular barriers [44,45,46]. Synthesis approaches are well established; spherical AuNPs typically use citrate (Turkevich) reduction while anisotropic shapes (nanorods, nanoshells) employ seed-mediated growth or template methods, after which surfaces are routinely PEGylated or conjugated to peptides/antibodies (e.g., targeting peptides) to improve circulation, reduce opsonization, and enhance tumor targeting [47].

In the context of orthotopic and xenograft models of PDAC, the administration of targeted AuNP formulations, when coupled with NIR irradiation, resulted in expedited intratumoral thermal elevation and significant reductions in tumor volume, with certain investigations documenting an inhibition of tumor proliferation of up to approximately 90% for the optimized AuNR-nanoplex in conjunction with light treatment protocols [47]. AuNPs have also been utilized as carriers for the delivery of chemosensitizing agents or molecular inhibitors such as resveratrol conjugates or KRAS-targeting agents to promote apoptotic mechanisms and reduce metastatic spread when applied in combination with PTT or chemotherapy in PC experimental models [42]. Significant translational barriers persist: the heterogeneous and fibrotic stroma of PDAC impedes efficient NP penetration, thereby limiting tumor cell delivery, while the prolonged biodistribution and clearance issues associated with non-biodegradable AuNPs pose safety and regulatory challenges that require resolution prior to widespread clinical application [48].

#### 5.1.2. Silver NPs

Silver nanoparticles (AgNPs) have gained attention as a promising therapeutic platform for PC owing to their preferential cytotoxicity toward PDAC cells while sparing normal tissue [49]. Evidence indicates that AgNPs activate multiple programmed cell death pathways, including apoptosis, autophagy, necroptosis, and mitotic catastrophe, in a manner dependent on particle size and concentration [49]. Mechanistically, AgNPs induce paraptosis-like cell death through mitochondrial dysfunction, endoplasmic reticulum (ER) dilation, and ROS accumulation, accompanied by upregulation of autophagic markers such as LC3b and p62 [50]. This paraptotic mechanism has been corroborated in vivo, where AgNP treatment markedly suppressed PDAC xenograft tumor growth with minimal systemic toxicity [50]. Extending these mechanistic insights, immunoglobulin G-functionalized silver nanoparticles have emerged as a promising photothermal therapy modality for pancreatic cancer by inducing Golgi apparatus dysfunction that activates the caspase-3 apoptotic cascade, thereby integrating targeted delivery with photothermal-mediated cytotoxicity [51]. Additional evidence implicates oxidative and nitro-oxidative stress as central drivers of AgNP-mediated cytotoxicity in pancreatic cancer, with PANC-1 cells exhibiting mitochondrial membrane disruption, cell cycle arrest, and reduced antioxidant capacity through depletion of enzymes such as SOD and GPX following exposure [52]. Notably, co-administration of α-lipoic acid selectively protected nonmalignant cells from AgNP-induced cytotoxicity while maintaining potent cytotoxic and antitumor activity against PDAC, thereby suggesting a viable strategy to enhance therapeutic selectivity [53].

#### 5.1.3. Iron-Oxide NPs

Superparamagnetic iron oxide nanoparticles (SPIONs) have been evaluated in PDAC as theragnostic agents, enhancing MRI contrast while enabling magnetic hyperthermia [54]. Upon exposure to alternating magnetic fields, SPIONs generate localized hyperthermia that facilitates tumor ablation and potentiates chemo- and radio sensitization in preclinical models [55]. Although these studies report significant tumor regression and increased cytotoxicity, clinical translation is constrained by pancreatic tumor depth and anatomical location, which impede therapeutic heating while posing risks of off-target damage [56]. Building on SPION-based strategies, ultra-fine iron oxide nanoparticles (uIONPs) encapsulating SN38 achieved receptor-mediated targeting and potent cytotoxicity in pancreatic cancer cells, with IC_50_ values of 11.8 ± 2.3 nM in MiaPaCa-2 and 20.8 ± 3.5 nM in Panc02 cells [57].

SPIONs are typically synthesized via co-precipitation of Fe^2+^/Fe^3+^ salts or thermal decomposition and stabilized with biocompatible coatings such as dextran, Polyethylene glycol (PEG), silica, or polymers to prevent aggregation and enable ligand or drug conjugation [57]. Surface functionalization with antibodies, peptides, or small molecules enhances tumor retention and selective uptake in PDAC models, supporting integrated imaging, targeted therapy, and magnetically triggered payload release [57]. Preclinical studies in PC demonstrate that intratumoral or locally administered SPIONs with AMF markedly decrease tumor cell viability and tumor burden in vitro and in vivo, while magnetic fluid hyperthermia enhances perfusion and drug uptake, thereby sensitizing tumors to chemotherapy or radiotherapy [58].

### 5.2. Carbon-Based NPs

#### 5.2.1. Graphene & Graphene-Oxid Platforms

Graphene-based nanomaterials, such as graphene oxide (GO) and reduced graphene oxide (rGO), are explored in PDAC as multifunctional platforms, offering high surface area for drug loading, strong near-infrared (NIR) absorbance for PTT, and facile surface modification for targeted delivery [26]. GO derivatives have been employed to deliver chemotherapeutics (e.g., gemcitabine, irinotecan analogues) and biologics while simultaneously serving as NIR photothermal transducers, enabling chemo-photothermal combination strategies in pancreatic cancer models [26]. Functionalization of GO with PEG, peptides, antibodies, or hyaluronidase improves colloidal stability and enables selective targeting of CD44/hyaluronan-rich stroma, while also locally modulating desmoplasia; together, these modifications enhance nanoparticle penetration and therapeutic delivery within PDAC tissue [58].

Recent work using GO composites in organoid and ex vivo PDAC models shows that engineered GO matrices enhance drug penetration and function as diagnostic or prognostic nanoplatforms through protein-corona fingerprinting [59]. Preclinical studies of rGO-mediated NIR PTT in orthotopic pancreatic tumor models have shown pronounced tumor heating and growth inhibition when combined with systemic chemotherapy, though cross-study comparisons remain limited due to variability in particle size, surface coating, dosage, and irradiation parameters [60].

#### 5.2.2. Carbon Nanotubes

Carbon nanotubes (CNTs), including single-walled (SWCNTs) and multi-walled (MWCNTs), have been widely investigated in PC due to their strong NIR absorbance for PTT, large surface area for drug or gene loading, and needle-like geometry that enhances tumor penetration [61]. PEGylated MWCNTs applied PANC-1 cells induced potent photothermal cytotoxicity under 808 nm laser irradiation (2 W), leading to mitochondrial membrane depolarization, ROS generation, and caspase-dependent apoptosis, thereby establishing a mechanistic basis for CNT-mediated nanophotothermolysis in PDAC [62]. Furthermore, CNT platforms have served as carriers for chemotherapeutics (e.g., paclitaxel, gemcitabine prodrugs), photosensitizers, and nucleic acids, enabling multimodal strategies such as chemo-PTT, chemo-PDT, and gene-silencing with thermal ablation, which have demonstrated synergistic antitumor efficacy in vitro and in xenograft models [61].

Multiple CNT-based studies report enhanced intracellular delivery and reduced IC_50_ values of chemotherapeutics in PDAC cell lines upon CNT conjugation, underscoring improved cellular uptake and retention as mechanistic drivers of efficacy [61].Consistently, the PDAC CNT literature highlights multifunctional CNT hybrids such as thermos-responsive hydrogel- or nanogel-embedded systems that improve tumor retention, permit localized intratumoral activation, and reduce systemic exposure, with these approaches undergoing validation in preclinical animal models [63,64].

#### 5.2.3. Carbon Quantum Dots & Graphene Quantum Dots

Graphene quantum dots (GQDs) and carbon quantum dots (CQDs) are sub-10 nm carbon nanomaterials with intrinsic fluorescence, tunable surface chemistry, and distinct photophysical properties that have been applied for both imaging and phototherapy in pancreatic cancer models [65]. Conjugation of GQDs with targeting ligands such as hyaluronic acid or RGD peptides enables selective binding to CD44- or integrin-overexpressing pancreatic cancer cells, thereby enhancing intratumoral accumulation and cellular internalization [66,67]. Under NIR irradiation, GQDs function as potent photothermal transducers, generating localized hyperthermia that induces membrane disruption, mitochondrial dysfunction, and tumor cell death in PANC-1 and other PDAC cell lines [68]. In orthotopic and spheroid models, GQD-mediated PTT has demonstrated superior therapeutic responses compared with chemotherapy alone and exhibited synergistic effects with systemic agents, resulting in reduced tumor growth and enhanced apoptosis when appropriately dosed and irradiated [68].

Researchers have explored CQDs and GQDs in PDAC as dual-function nanoplatforms that combine fluorescence-based imaging with phototherapy. In PC cell models, CQDs generate ROS upon light activation, driving ROS-dependent cell death and enhancing photodynamic therapy (PDT) efficacy [60]. Hybrid systems such as GQD–hyaluronic acid conjugates loaded with chemotherapeutics improve delivery to PANC-1 cells, enabling reduced systemic doses while preserving or enhancing antitumor activity [62]. Surface modifications, including PEGylation, albumin conjugation, and polysaccharide coatings, improve colloidal stability and tumor targeting in PDAC models by prolonging circulation and reducing reticuloendothelial uptake [69]. For instance, albumin–GQD constructs have been shown to facilitate uptake by PANC-1 cells and enable fluorescence-guided drug delivery [70].

Preclinical PDAC studies demonstrate that GQDs act as efficient photothermal transducers under near-infrared irradiation, inducing dose- and depth-dependent hyperthermia in spheroid and orthotopic models [71]. Multiparametric analyses confirm that GQD-mediated photothermal therapy triggers apoptosis, necrosis, and immunogenic cell death, depending on surface chemistry and irradiation parameters [71]. In vivo, systemically delivered GQD conjugates accumulate in pancreatic tumors, enabling image-guided therapy, while local NIR irradiation elevates tumor temperature and suppresses tumor growth compared to controls [71].

#### 5.2.4. Nanodiamonds

Nanodiamonds (NDs) are sp^3^-carbon NPs with tunable surface chemistry that supports PEGylation and drug conjugation, making them attractive platforms for tumor-selective drug delivery and stromal modulation in PDAC [72]. In immune-competent genetically engineered mouse models of PDAC, PEGylated NDs co-loaded with irinotecan and curcumin produced marked antitumor responses, reduced metastatic dissemination, and downregulated immunosuppressive cytokines such as IL-10, indicating both direct cytotoxic and tumor–microenvironmental effects [73].

Mechanistic investigations demonstrate that NDs improve intratumoral retention and penetrate stromal barriers, enabling the simultaneous delivery of hydrophobic and hydrophilic agents and supporting combinatorial regimens in settings where monotherapies are inadequate [72]. Furthermore, ND-based platforms mitigate drug resistance by enhancing intracellular drug accumulation and permitting effective tumor control at reduced cytotoxic doses, thereby potentially lowering systemic toxicity, an effect substantiated in preclinical PDAC efficacy studies [74].

### 5.3. Lipid-Based NPs

#### 5.3.1. Solid Lipid Nanoparticles

Solid lipid nanoparticles are biocompatible carriers composed of physiological lipids that have emerged as promising platforms for PC therapy [75]. Compared to inorganic or polymeric nanoparticles, SLNs exhibit markedly lower risks of acute and chronic toxicities while enhancing circulation time, permeability, and the bioavailability of frontline chemotherapeutics [76]. SLNs can be prepared by high-pressure homogenization, cold homogenization, or solvent emulsification, allowing efficient encapsulation of both hydrophilic and lipophilic agents within the lipid matrix [77]. Notably, herniarin-loaded SLNs demonstrated strong cytotoxic activity against Panc-1 pancreatic cancer cells, with an IC_50_ of 83.744 µL, underscoring their therapeutic potential [77].

#### 5.3.2. Nanostructured Lipid Carriers

Nanostructured lipid carriers (NLCs) are second-generation lipid NPs composed of both solid and liquid lipids, forming an unstructured matrix that allows greater drug loading and improved long-term stability compared to conventional SLNs [78]. These systems provide distinct advantages, including enhanced solubility, permeability, bioavailability, storage stability, reduced systemic toxicity, prolonged half-life, and targeted tissue delivery [79].

Targeted NLCs incorporating a gemcitabine–stearic acid prodrug and hyaluronic acid–baicalin conjugate demonstrated superior cytotoxicity against AsPC-1 pancreatic cancer cells and pronounced antitumor efficacy in murine models [79]. With encapsulation efficiencies of 85–95%, particle sizes of 100–300 nm, and sustained release over 24 h, these formulations highlight the dual role of hyaluronic acid as both a targeting ligand and a therapeutic component [78].

### 5.4. ADAM9-Responsive Mesoporous Silica NPs

PDAC is a highly aggressive malignancy with a poor prognosis, in large part because systemic chemotherapy is restricted by severe dose-dependent toxicities and limited tumor specificity [80]. To overcome these limitations, mesoporous silica nanoparticles (MSNs) have been engineered as targeted drug delivery systems that exploit A Disintegrin and Metalloproteinase 9 (ADAM9), a protease markedly enriched in PDAC relative to normal tissues and circulating cells [80].

The ADAM9-responsive MSN platform operates through a gatekeeping mechanism in which drug-loaded nanoparticles are capped with avidin via a peptide linker containing an ADAM9-cleavable sequence [80]. In the PDAC microenvironment, tumor-secreted ADAM9 cleaves this linker, displacing the avidin cap and releasing chemotherapeutics such as paclitaxel directly at the tumor site [81]. This release does not occur in the presence of blood proteases or leukocyte-derived media, ensuring high specificity [82]. In vitro studies demonstrated that paclitaxel-loaded MSNs selectively induced PDAC cell death while sparing nonmalignant cells [80]. These findings establish ADAM9 as a viable biomarker for targeted therapeutic strategies in PDAC [80]. By leveraging protease-responsive linkers, MSN-based systems achieve spatially controlled drug release, effectively enhancing antitumor activity while minimizing systemic toxicity. This approach illustrates the potential of rationally designed nanocarriers to address long-standing challenges in pancreatic cancer treatment [83].

### 5.5. Polymeric Micelles

Polymeric micelles are nanoscale drug delivery systems engineered to improve chemotherapeutic delivery to pancreatic tumors, overcoming the limitations of conventional regimens [31].These core–shell structures, typically <100 nm, are formed by the self-assembly of amphiphilic block copolymers in aqueous environments [31]. Hydrophobic drugs are sequestered within the micelle core, while the hydrophilic corona, often composed of PEG, enhances stability and circulation by minimizing protein adsorption reported as 4.5-fold lower than in non-PEGylated liposomes [84]. Their small size promotes preferential tumor accumulation via the EPR effect, a phenomenon exploited in PC models [84].

Among the different NP platforms discussed in this review, metallic NPs, particularly gold, silver, selenium, and iron oxide NPs, appear to be highly compatible with green synthesis approaches due to their ability to be generated using plant extracts or biological systems that act as reducing and stabilizing agents [85]. These platforms can benefit from the incorporation of phytochemical-derived surface functionalities, potentially enhancing their biological activity and biocompatibility [84].

Lipid-based and polymeric nanocarriers may also benefit from green approaches using naturally derived materials such as chitosan, alginate, and plant-derived components, which can improve biodegradability and reduce toxicity. However, the advantages of green synthesis are not uniform across all NP classes and depend on factors including NP composition, synthesis method, biological source, and intended therapeutic application. Therefore, green technology should be considered a strategy to optimize specific nanoplatforms rather than a universally superior approach for all NP systems [85].

A representative system is DACHPt/m, in which the platinum agent (1,2-diaminocyclohexane)platinum(II) forms polymer–metal complexes with PEG–poly(glutamic acid) block copolymers [84]. Surface modification with anti-tissue factor (TF) antibody fragments yields immunomicelles (anti-TF Fab’-DACHPt/m) that exhibit enhanced binding and uptake in TF-overexpressing pancreatic cancer cells. This results in superior cytotoxicity compared with non-targeted micelles, and the acidic, chloride-rich endosomal environment facilitates intracellular drug release [84]. In vivo studies demonstrated that Fab’-functionalized DACHPt/m micelles achieved sustained tumor suppression in pancreatic xenografts for more than 40 days, outperforming free DACHPt and untargeted micelles [84]. These findings underscore the capacity of polymeric micelles to integrate passive targeting through the EPR effect with active targeting via antibody conjugation, thereby increasing intratumoral drug concentrations while minimizing systemic toxicity [84]. Despite the promising potential of different nanoplatforms, their application in herbal-based PDAC therapy remains associated with several challenges. Many reported nanoparticle systems are evaluated using isolated phytochemicals or conventional drugs rather than standardized herbal extracts, limiting direct clinical translation. Moreover, variations in plant composition, NP synthesis parameters, and incomplete pharmacokinetic characterization remain major obstacles. Therefore, future studies should focus on standardized green synthesis protocols and comprehensive evaluation of herbal nanoformulations to facilitate clinical development.

## 6. Green Synthesis of Nanomaterials

Green synthesis represents an environmentally sustainable approach for NP fabrication that utilizes biological resources, including plant extracts, microorganisms, and biopolymers, as reducing, stabilizing, or capping agents during NP formation [86]. Therefore, the term “green nanoparticles” should specifically describe nanomaterials produced through biological synthesis pathways rather than NPs that only incorporate herbal-derived compounds as therapeutic cargos. Compared with conventional physicochemical approaches, including chemical reduction, thermal decomposition, and physical vapor-based techniques, green synthesis minimizes the use of toxic solvents, hazardous reducing agents, and energy-intensive processes. Traditional synthesis methods may generate harmful by-products and produce NPs with limited biocompatibility, restricting their biomedical applicability [85,86]. In contrast, green synthesis is generally performed under mild aqueous conditions using renewable biological materials, reducing environmental impact while potentially improving NP safety and biological functionality [87,88].

Although herbal nanomedicine and green nanotechnology are closely related, they represent distinct concepts. Herbal nanomedicine focuses on the nanoscale delivery of plant-derived bioactive compounds to improve their therapeutic performance, whereas green nanotechnology refers primarily to the environmentally responsible synthesis of NPs. These approaches can overlap when plant extracts simultaneously provide bioactive molecules and function as reducing or stabilizing agents during NP fabrication.

Despite the increasing interest in green nanotechnology, it is important to distinguish its current developmental stage from clinically established nanomedicine platforms. Clinically approved systems, such as liposomal irinotecan and albumin-bound paclitaxel, have demonstrated improved pharmacokinetics, tumor accumulation, and therapeutic efficacy through validated delivery mechanisms. In comparison, green-synthesized NPs remain largely within the preclinical research stage. Their potential advantages include sustainable production, reduced reliance on toxic reagents, enhanced biocompatibility, and incorporation of biological activities derived from natural components; however, direct evidence demonstrating superiority over clinically validated non-green nanocarriers remains limited [89].

Conventional NP delivery systems can enhance the solubility, stability, and bioavailability of herbal compounds, whereas green-synthesized herbal nanoformulations may provide additional benefits by combining sustainable fabrication with the intrinsic biological activities of plant-derived molecules. Nevertheless, differences in plant composition, synthesis parameters, and limited pharmacokinetic and clinical investigations currently prevent definitive comparisons between green and conventional nanoformulations. Therefore, standardized synthesis protocols and clinically relevant models are required to clarify their therapeutic advantages [88].

**Figure 3:**
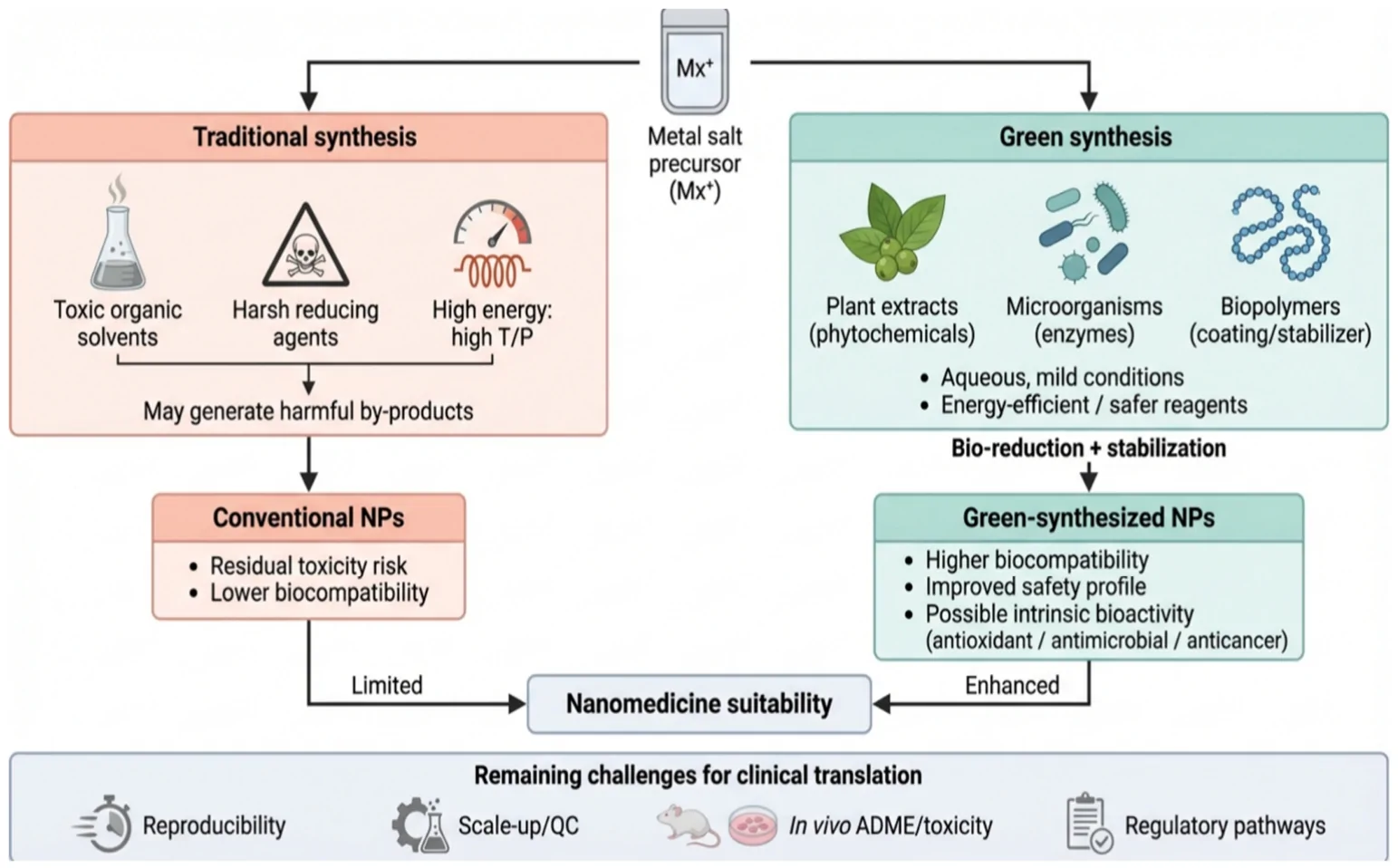
Graphical representation of the therapeutic landscape for PDAC. The left panel illustrates the limitations of conventional chemotherapy, where dense desmoplastic stroma and high interstitial fluid pressure (IFP) lead to poor drug penetration and resistance. The right panel highlights nanomedicine strategies, utilizing various nanoparticles (liposomal, polymeric, lipid-based) equipped with targeting ligands (e.g., CD44) and TME-responsive triggers (pH, redox, MMPs) to enhance tumor accumulation and therapeutic efficacy.

Green nanotechnology marks a transformative shift in how nanomaterials are synthesized, embedding sustainability and environmental responsibility at the core of biomedical innovation. Drawing from the principles of green chemistry, this approach emphasises maximising atom efficiency, employing non-toxic solvents, utilizing energy-efficient methods, and replacing hazardous chemical reducers with safer alternatives. Such strategies not only reduce ecological impact but also enhance the biocompatibility of NPs, a factor of critical importance in therapeutic applications [90]. Recent literature emphasizes that green synthesis enables the production of metal and metal oxide NPs with well-defined shapes, lower toxicity, and promising uses in areas such as drug delivery and tissue engineering. By merging biology and materials science, green nanotechnology supports the development of multifunctional nanomaterials that are both therapeutically relevant and consistent with international sustainability objectives. This integration effectively links environmentally responsible design with practical clinical applications [91]. The therapeutic landscape of PDAC and the transition from conventional chemotherapy toward nanomedicine-based therapeutic strategies are summarized in Figure 3.

### 6.1. Biological Sources and Mechanisms of Green Nanoparticle Synthesis

Biological sources used for green NP synthesis include plants, microorganisms, and naturally derived polymers. These biological systems provide diverse biomolecules capable of reducing metal ions, stabilizing NP structures, and influencing their physicochemical properties. Plant extracts are among the most widely investigated biological platforms because they contain abundant phytochemicals, including polyphenols, flavonoids, terpenoids, alkaloids, and saponins. These compounds can act as reducing and stabilizing agents, facilitating NP formation under mild conditions while contributing additional biological activities, including antioxidant, antimicrobial, and anticancer effects [91].

The biological functionality of plant-mediated NPs is not solely attributed to the green synthesis process itself but also to the biochemical characteristics of the phytochemicals associated with NP surfaces. Surface-bound molecules may influence colloidal stability, cellular uptake, ROS generation, and anticancer responses through synergistic interactions between phytochemical components and the NP core. Furthermore, NP characteristics, including particle size, surface charge, and functional groups, can affect biodistribution, cellular internalization, and interactions with tumor-associated components. However, potential targeting effects mediated by plant-derived surface molecules remain context-dependent and require experimental validation rather than being considered universal properties of green NPs [92].

Microorganisms, including bacteria, fungi, algae, and actinomycetes, also serve as biological “nano-factories” by producing enzymes capable of reducing metal ions and stabilizing NP structures. Microbial-mediated synthesis may occur extracellularly, simplifying downstream processing and offering potential advantages for scalability. Enzyme-driven mechanisms, including nitrate reductase-mediated pathways, enable NP formation under mild conditions with favorable uniformity, stability, and biocompatibility. Nevertheless, challenges such as sterile cultivation requirements, variable production yields, and difficulties in achieving reproducible manufacturing standards remain barriers to clinical translation [90,91,92,93].

Biopolymers such as chitosan, cellulose, alginate, and PHAs provide effective stabilization and functional coatings for NPs. Their biodegradable nature allows precise control over particle morphology while improving biocompatibility, making them highly suitable for applications in drug delivery and tissue engineering [92]. In one recent study, chitosan NPs were generated using *Blumea balsamifera* leaf extract under gentle, green synthesis conditions, producing spherical particles with strong antibacterial effects, illustrating the combined benefits of biopolymer frameworks and phytochemical agents [93].

### 6.2. Biological Advantages and Therapeutic Potential of Green-Synthesized Nanomaterials

Green nanotechnology represents an important integration between biological systems and materials science, aiming to produce multifunctional nanomaterials with improved sustainability and biomedical compatibility. By following the principles of green chemistry, these approaches emphasize the use of renewable resources, non-toxic solvents, energy-efficient processes, and safer alternatives to conventional chemical reducing agents. Such strategies reduce environmental impact while potentially improving NP biocompatibility and therapeutic applicability [89].

Recent studies have demonstrated that green synthesis can generate metal and metal oxide NPs with controlled physicochemical properties, including defined morphology, reduced toxicity, and potential applications in drug delivery, tissue engineering, and biomedical technologies. The combination of biological components with nanomaterial engineering provides opportunities for developing multifunctional platforms that integrate environmental sustainability with therapeutic functions [90].

The biological activity of green-synthesized NPs is influenced not only by the synthesis approach but also by the phytochemical composition of the biological sources involved. Plant-derived molecules, including polyphenols, flavonoids, terpenoids, and alkaloids, may remain associated with NP surfaces after synthesis, contributing to enhanced biological interactions. These surface-associated compounds can affect NP stability, cellular uptake, ROS modulation, and anticancer activity through combined effects between the biological coating and NP core [91].

Furthermore, the physicochemical characteristics of green-synthesized NPs, including particle size, surface charge, and surface functional groups, represent critical determinants of their biological performance. These parameters influence biodistribution, cellular internalization, and interactions with tumor-associated components. Although plant-derived surface molecules may contribute to preferential interactions with cancer cells in specific models, such effects remain dependent on NP composition, biological context, and experimental validation [92].

### 6.3. Current Challenges Limiting Clinical Translation of Green Nanomedicine

Despite the considerable advantages of green-synthesized nanomaterials, their translation from laboratory research to clinical applications remains challenging. Unlike conventional synthetic approaches, biological synthesis depends on natural sources with inherent variability in chemical composition. Differences in plant species, cultivation conditions, extraction procedures, or microbial strains may influence nanoparticle size, morphology, surface characteristics, and biological activity, resulting in batch-to-batch variation [94].

In addition, although many green-synthesized NPs demonstrate favorable biocompatibility profiles in preliminary studies, comprehensive investigations addressing long-term toxicity, biodistribution, pharmacokinetics, immune responses, and clearance mechanisms remain limited. These factors are essential for evaluating safety and therapeutic feasibility before clinical application.

Large-scale production also represents a major challenge, as industrial manufacturing requires strict control of biological sources, synthesis parameters, purification procedures, and quality assurance processes. Achieving Good Manufacturing Practice (GMP)-compatible production while maintaining NP reproducibility and biological activity remains a critical requirement for successful translation. Therefore, advancing green nanomedicine toward clinical use requires multidisciplinary integration of nanotechnology, toxicology, pharmacology, and regulatory sciences [94].

Compared with traditional chemical and physical synthesis methods, green approaches provide several advantages, including reduced use of toxic reagents, elimination of extreme processing conditions, and utilization of renewable biological resources. These characteristics improve sustainability and may enhance the biological functionality of NPs by retaining natural compounds. However, the assumption that green-synthesized NPs are universally superior to conventional systems should be avoided, as therapeutic performance depends on NP composition, formulation parameters, and application context [89,91,95,96].

### 6.4. Proposed Standardization Framework for Green-Synthesized Nanomaterials

To facilitate the clinical development of plant- and microorganism-mediated nanomaterials, standardized quality-control frameworks are required to ensure reproducibility, safety, and consistent biological performance. First, authentication of biological sources should be performed through appropriate taxonomic identification, genetic barcoding when applicable, and comprehensive phytochemical or microbial profiling to define the active reducing and stabilizing components.

Second, carefully control and report critical synthesis parameters, including biological extract concentration, precursor ratio, pH, temperature, reaction duration, and purification procedures, to ensure reproducible NP production. Third, comprehensive physicochemical characterization should include particle size distribution and polydispersity index assessment using dynamic light scattering, morphology evaluation through transmission or scanning electron microscopy, surface charge analysis by zeta potential measurements, structural characterization using X-ray diffraction (XRD), and chemical composition analysis through techniques such as Fourier-transform infrared spectroscopy, Raman spectroscopy, X-ray photoelectron spectroscopy, or elemental analysis [97].

Furthermore, reproducibility assessment should involve comparison of multiple independent production batches using predefined acceptance criteria related to particle size, morphology, surface chemistry, loading efficiency, and biological activity. Functional evaluation should include standardized assays for cytotoxicity, cellular uptake, immune compatibility, and pharmacological effects. Finally, implementation of GMP-compatible manufacturing processes and appropriate regulatory documentation will be essential before advancing green-synthesized nanomaterials toward clinical trials [98].

## 7. Green Synthesized Nanoparticles Based on Herbs for Pancreatic Cancer Treatment

Green synthesis of NPs employs plant extracts as reducing and stabilizing agents, replacing conventional chemical methods with an eco-friendly approach [94]. Phytochemicals such as polyphenols, flavonoids, alkaloids, and terpenoids mediate NP formation while conferring additional therapeutic functionalities [95]. The resulting nanostructures generally demonstrate enhanced biocompatibility, lower toxicity toward normal cells, and synergistic anticancer potential arising from the retained bioactive plant constituents [96]. In PC, green-synthesized NPs offer solutions to key therapeutic challenges, including poor bioavailability of many natural compounds, lack of tumor specificity in systemic chemotherapy, and dose-limiting toxicities [96]. These NPs exert cytotoxicity in PDAC cells through diverse mechanisms, including ROS generation, apoptosis induction, cell cycle arrest, and interference with vital cellular pathways [98,99].

Preclinical studies have reported promising outcomes using plant-mediated silver, gold, copper oxide, iron oxide, zinc oxide, and selenium nanoparticles against PDAC cell lines such as PANC-1, AsPC-1, and MIA PaCa-2 [100,101,102,103]. These NPs not only exhibit direct cytotoxic effects but also enhance the efficacy of conventional chemotherapeutic agents when used in combination therapy. Recent studies have explored the potential of herbal-derived NPs synthesized via green methods for PC management, highlighting their therapeutic promise and limitations, as shown in Table 2.

### Clinical Limitations and Unresolved Challenges of Herbal-Based Nanomedicine

Despite the promising anticancer potential of herbal-derived compounds and their nanoformulated derivatives, several unresolved issues must be considered before clinical implementation. Herb–drug interactions represent a critical concern, particularly in oncology patients receiving combination chemotherapy. Phytochemicals may influence drug absorption, metabolism, transport pathways, or therapeutic efficacy through modulation of enzymes such as cytochrome P450 isoforms and drug transporters. Therefore, comprehensive pharmacokinetic evaluation and interaction studies are required before combining herbal nanomedicines with standard anticancer therapies [111].

In addition, reported benefits of herbal products should be interpreted cautiously, as traditional use and patient-reported improvements may reflect placebo effects and cannot substitute for evidence from controlled clinical studies. Furthermore, the complex chemical composition of botanical extracts often limits mechanistic understanding, making it difficult to identify the specific bioactive components responsible for therapeutic effects. Advanced analytical approaches, including metabolomics, molecular profiling, and target validation studies, are required to clarify mechanisms of action and support rational clinical development [112].

## 8. Delivery Systems of Herbs for Pancreatic Cancer

To improve therapeutic targeting and bioavailability, researchers have thoroughly examined a variety of nanomaterial-based drug delivery devices for the delivery of Traditional Chinese Medicine bioactive substances [87]. Compared with conventional treatment modalities, the high efficacy and safety of targeted delivery systems enabled by loading pharmaceuticals onto NPs constitute a major advantage of nanotechnology. Furthermore, antioxidant activity, protection of volatile components, prevention of enzymatic degradation, and pH-dependent release management are key ways nanomaterial-encapsulated drug delivery systems can mitigate environmental effects on medications both in vivo and in vitro. Certain nanomedicines, such as the albumin-bound abiraterone acetate crystal formulation and others, are currently authorised for the therapeutic treatment of prostate cancer [113].

One of the primary challenges in translating plant-derived anticancer compounds into clinical therapy is their poor aqueous solubility, rapid metabolism, and limited systemic bioavailability. Several phytochemicals with promising anticancer properties against pancreatic cancer, including curcumin, resveratrol, and other polyphenols, reach low plasma concentrations after oral administration because of rapid biotransformation and limited absorption. For example, clinical observations have shown that oral doses of 8–12 g of curcumin produce only nanogram-level plasma concentrations, demonstrating the limitations of conventional delivery strategies. Nanotechnology-based delivery systems have therefore been investigated to enhance the pharmacokinetic profiles of these compounds by improving solubility, protecting the bioactive molecules from metabolic degradation, and facilitating tumor-specific accumulation through passive or active targeting mechanisms [114].

Nanoparticle-mediated delivery systems can exploit the EPR effect, a phenomenon in which NPs preferentially accumulate in tumor tissue due to the abnormal vasculature and impaired lymphatic drainage characteristic of solid tumors such as pancreatic ductal adenocarcinoma [115]. In addition to passive targeting through EPR, NPs can be functionalized with specific ligands that recognize receptors overexpressed on pancreatic cancer cells. For instance, transferring receptors are frequently overexpressed in pancreatic cancer due to the increased iron requirements of rapidly proliferating tumor cells. NPs conjugated with transferrin or similar targeting ligands can therefore enhance the cellular uptake of encapsulated phytochemicals and improve the specificity of drug delivery to malignant cells [114].

Encapsulating phytochemicals in nanocarriers has also been shown to improve therapeutic efficacy by enhancing intracellular delivery and controlled drug release. A notable example is resveratrol-loaded gold nanoparticles, which have shown improved stability and delivery efficiency compared with free resveratrol. In pancreatic cancer models, these nanoparticles increased cell-cycle arrest and apoptosis in PANC-1 cells while reducing nonspecific cytotoxicity in normal macrophages. Moreover, the nanoparticle formulation exhibited pH-responsive drug release behavior, facilitating enhanced drug accumulation within the acidic tumor microenvironment and thereby improving antitumor activity [116].

Other nanoformulation strategies have also been explored to further enhance the anticancer activity of herbal compounds. Magnetic nanoparticle-based delivery systems, for example, have been used to encapsulate resveratrol within nanocarriers that enable improved cellular internalization and targeted accumulation in pancreatic cancer cells. In vitro studies using Capan-1 pancreatic cancer cells demonstrated that resveratrol-loaded magnetic nanocarriers significantly increased cytotoxicity compared with free resveratrol and promoted the expression of pro-apoptotic genes such as Bax, Fas, and p53, while suppressing anti-apoptotic signalling pathways. These findings indicate that nanocarrier-based delivery can potentiate the therapeutic activity of phytochemicals by enabling efficient intracellular drug transport and modulating cancer-related signaling pathways [117,118].

Beyond improving the bioavailability of individual phytochemicals, nanotechnology platforms enable co-delivery strategies that combine plant-derived compounds with conventional chemotherapeutic agents to improve treatment outcomes. Curcumin-loaded nanoparticles, for instance, have been investigated in combination with gemcitabine in orthotopic pancreatic tumor models, where the nano formulation significantly inhibited tumor growth and metastasis compared with either treatment alone. These synergistic effects are attributed to the ability of curcumin to modulate multiple oncogenic signalling pathways, including NF-κB, STAT3, and PI3K/AKT, while the nanoparticle carrier ensures sustained drug release and improved tumor accumulation [114].

To address the pharmacokinetic and therapeutic limitations of many plant-derived anticancer compounds, researchers have developed and evaluated numerous nanotechnology-based delivery platforms in pancreatic cancer research. These nanoformulations vary in composition, size distribution, and targeting strategies, which can significantly influence cellular uptake, biodistribution, and therapeutic performance. Several studies have therefore investigated different NP systems for delivering herbal bioactive compounds, assessing parameters such as particle size, phytochemical origin, experimental models used, and resulting antitumor outcomes. A summary of representative nanotechnology-based delivery systems reported for plant-derived compounds targeting pancreatic cancer, including their NP type, particle size range, phytochemical source, experimental models, and key findings, as shown in Table 3.

## 9. Herbzyme for Pancreatic Cancer

Herb-derived enzymes refer to enzyme-based therapeutic systems or nanozyme platforms that originate from plant-derived materials, plant-associated biomolecules, or are fabricated using herbal components as functional or catalytic elements. This concept should be distinguished from conventional nanozymes that possess enzyme-like activities but have no direct association with herbal sources or green synthesis approaches. Based on their origin and synthesis strategy, nanozyme systems discussed in this section are classified into three categories: (i) plant-derived or herbal-assisted nanozymes, in which plant components contribute to NP formation or catalytic activity; (ii) green-synthesized nanozymes produced using biological reducing or stabilizing agents; and (iii) non-herbal nanozymes with enzyme-mimicking properties that provide mechanistic insights but are not considered herb-derived systems.

**Figure 4:**
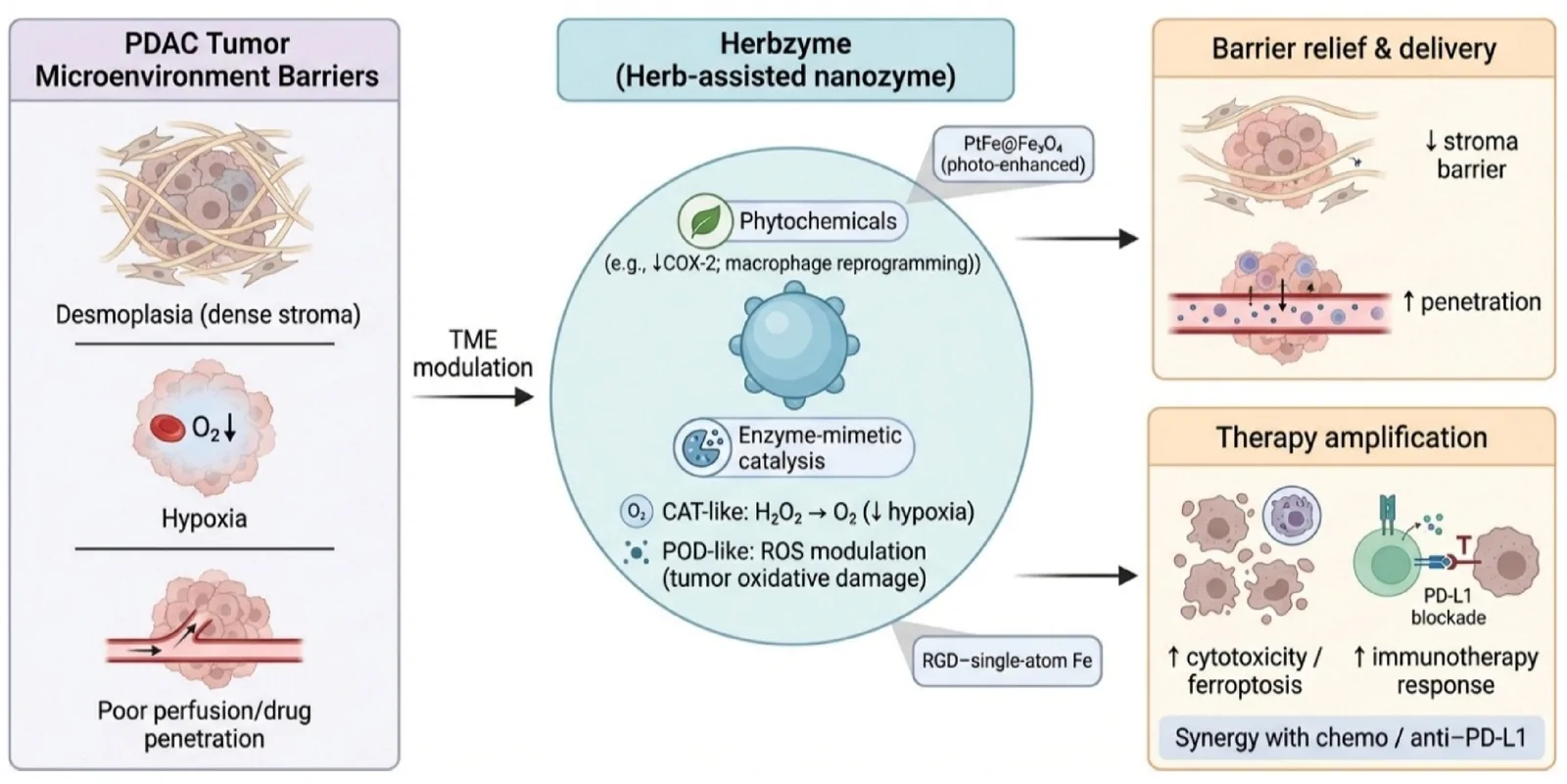
Herbzyme-mediated remodelling of the PDAC TME to enhance therapeutic efficacy. The figure illustrates the mechanisms by which herbzyme-based nanoplatforms modulate the TME, including stromal remodeling, immune regulation, and enhanced therapeutic delivery. The arrows indicate the directional interactions and sequential processes involved in TME modulation and therapeutic responses.

Recent advancements in nanozyme technology have led to the emergence of herbzymes plant-derived or herb-assisted nanozymes that integrate the intrinsic bioactivity of phytochemicals with enzyme-mimetic catalysis. This hybrid functionality presents a promising avenue for modulating the complex tumor microenvironment of PDAC, characterized by dense desmoplasia, oxidative stress, and poor vascularization [126]. Recent evidence suggests that nanozyme-based systems can overcome the stromal and hypoxic barriers of PDAC, thereby improving drug penetration and therapeutic efficacy. For instance, a photo-enhanced PtFe@Fe_3_O_4_ nanozyme demonstrated peroxidase- and catalase-like activity, effectively alleviating tumor hypoxia and enhancing cytotoxicity in deep pancreatic tumors [100]. Moreover, an RGD-functionalized single-atom iron nanozyme triggered ferroptosis and potentiated anti-PD-L1 immunotherapy in orthotopic PDAC models [127]. Complementarily, herbal formulations have shown significant bioactivity against PDAC, as *Anchusa* strigosa extract suppressed COX-2 expression, cell proliferation, and migration in PDAC cells [128], while the traditional Japanese Kampo medicine Juzentaihoto enhanced survival and reprogrammed macrophage polarization in gemcitabine-treated mice [129]. Collectively, these findings underscore the potential of herbzyme-based therapeutics as redox modulators, stromal-penetrating nanocarriers, or immune-adjuvant systems in PDAC therapy and warrant further mechanistic and translational investigation, as illustrated in Figure 4.

## 10. Challenges and Limitations

Green synthesis of NPs utilizing biological sources such as plants, microbes, and biomolecules represents a sustainable, cost-efficient, and environmentally benign alternative to conventional chemical routes. Despite these advantages, significant challenges remain regarding reproducibility, scalability, and precise control of NP physicochemical properties. A key limitation arises from the intrinsic variability of biological precursors including plant extracts and microbial cultures, which often results in batch-to-batch heterogeneity in NP size, morphology, and composition. These inconsistencies largely stem from fluctuations in phytochemical profiles, seasonal influences, and the growth conditions of the biological material. Moreover, fine-tuning critical environmental parameters, including pH, temperature, and ionic strength, is essential for tailoring NP characteristics, yet precise regulation under green synthesis conditions remains technically difficult. These limitations pose barriers to large-scale manufacturing and hinder the standardization required for clinical translation and commercial deployment of green-synthesized nanomaterials [130].

The mechanistic roles of phytochemicals such as phenolics, terpenoids, alkaloids, and proteins in reducing metal ions and stabilizing NPs remain incompletely understood. Understanding how these biomolecules interact with metallic precursors is critical for controlled nucleation and growth, ensuring uniform particle size and morphology. Plant-mediated synthesis, often carried out under mild conditions, frequently produces NPs with amorphous or semi-crystalline structures, which complicate precise characterization using standard techniques such as XRD. This variability further challenges reproducible synthesis of NPs with the well-defined crystalline features needed for biomedical applications. Additionally, biomolecular residues on NP surfaces strongly influence colloidal stability, biocompatibility, and biological activity, yet these surface chemistries can vary widely depending on the biological source and synthesis parameters [131].

Additional limitations of green NP synthesis include slower reaction kinetics and greater sensitivity to environmental parameters than conventional chemical methods [87]. Enzyme-mediated approaches, for instance, are expensive and require stringent control of temperature and pH to preserve catalytic activity, while reaction times are often longer than those of chemical protocols. Furthermore, substrate specificity and incompatibility with certain metal precursors limit the versatility and broader applicability of biosynthetic strategies. Industrial translation is also hindered by labour-intensive procedures and the absence of automation, resulting in low throughput and poor reproducibility, which collectively constrain large-scale production and clinical adoption [87].

Finally, critical challenges in green NP synthesis also relate to safety, cytotoxicity, and environmental implications. While these nanomaterials are often regarded as biocompatible, variations in size, morphology, and surface chemistry can lead to heterogeneous biological responses, underscoring the need for rigorous in vitro and in vivo toxicity evaluations. Equally important, the biodistribution, long-term accumulation, and ecological impact of bio-inspired NPs remain insufficiently characterized. The absence of standardized protocols for biological assessment, environmental monitoring, and regulatory validation continues to impede their safe translation into clinical, agricultural, and environmental applications [132,133,134].

Despite the promising therapeutic potential of herbal-based nanomedicine for PDAC, most current studies remain limited to in vitro cellular models and conventional xenograft mouse models. Although these approaches provide valuable insights into cytotoxicity, mechanism of action, and preliminary therapeutic efficacy, they do not fully recapitulate the complex heterogeneity, stromal architecture, and immune interactions of human PDAC. Patient-derived xenograft (PDX) models and patient-derived organoids represent more clinically relevant platforms that can better preserve tumor characteristics and drug response patterns. However, their application in herbal nanoformulation studies remains limited. Future investigations should prioritize the integration of advanced preclinical models, including PDX and organoid systems, to improve translational predictability and facilitate clinical development of green nanomedicine-based strategies for PDAC [134].

## 11. Future Perspectives

Emerging approaches in nanomedicine hold significant promise for overcoming the persistent challenge of drug resistance in PDAC. Future research should emphasize the design of multifunctional nanocarriers capable of simultaneously delivering diverse therapeutic agents such as chemotherapeutics, gene-silencing molecules, and stromal-modulating compounds in order to address both tumor-intrinsic and microenvironment-mediated mechanisms of resistance [133]. The forthcoming generation of advanced nanotherapeutics should be designed to incorporate stimuli-responsive features, enabling them to release their therapeutic payload in reaction to local cues such as pH fluctuations, redox imbalances, or specific enzymatic activities within the tumor milieu [28]. Moreover, strategies that actively remodel the tumor microenvironment through stromal breakdown or modulation of the vasculature will be essential to enhance therapeutic efficacy [28].

Despite promising advances in preclinical nanotherapeutics for PDAC, translation into clinical applications remains a significant challenge. Ensuring long-term efficacy and biocompatibility in humans is critical, as outcomes observed in cellular or animal models may not fully predict responses in the complex human tumor microenvironment [134]. Moreover, clinical translation requires scalable, environmentally sustainable manufacturing methods to ensure product quality and stability, as well as well-designed clinical protocols to assess safety, optimal dosing, and pharmacokinetics across diverse patient populations. Close multidisciplinary collaboration among nanotechnology researchers, oncologists, pharmacologists, and regulatory bodies is essential to overcome these obstacles and enable the safe and effective application of precision nanomedicine in pancreatic cancer treatment [28].

An important direction for the field is developing theragnostic systems that combine diagnostic imaging with therapeutic delivery, allowing clinicians to track treatment responses in real time and adjust interventions accordingly [28].Yet, bringing such advances into clinical use requires overcoming key obstacles, including the need for scalable and environmentally sustainable synthesis methods, assurance of long-term safety and biocompatibility, and navigation of complex regulatory frameworks [33]. Progress in this area will depend on close cross-disciplinary collaboration among nanotechnology researchers, oncologists, pharmacologists, and regulatory authorities to refine these strategies and pave the way for precise, sustainable nanotherapies in pancreatic cancer care [83].

## 12. Conclusions

Green nanomedicine is emerging as a promising paradigm for PC therapy by uniting traditional herbal pharmacology with innovative, sustainable nanotechnology. NPs derived from herbal sources enhance the solubility, stability, and delivery efficiency of bioactive molecules, while also enabling selective tumor targeting and minimizing systemic side effects. Unlike conventional nanomaterial synthesis, green approaches that employ plants, microorganisms, and natural polymers offer safer, more economical, and environmentally responsible alternatives. Although preclinical studies have shown encouraging outcomes, key hurdles remain, particularly in achieving large-scale reproducibility, ensuring long-term biosafety, and establishing clear regulatory frameworks. Future directions should prioritize refining synthesis methodologies, leveraging multi-omics tools to unravel underlying mechanisms, and advancing well-designed clinical trials to confirm therapeutic effectiveness. Addressing these challenges will strengthen the role of green nanotechnology as a sustainable and effective strategy for managing PC.

## Figures and Tables

**Table 1:** Advantages and disadvantages of herbal-derived compounds used for cancer therapy.

Herbal-Derived Compounds	Advantage	Disadvantage	Reference
**Polyphenols**	Because of their vast structural diversity, polyphenols have a variety of therapeutic effects, including antioxidant and anticancer properties.	Poor solubility and bioavailability, poor targeting ability, and limited stability restrict their use in the therapeutic treatment of malignancies.	[19,20,21]
**Alkaloids**	Numerous cancers, such as breast cancer, Hodgkin’s lymphoma, acute lymphoblastic leukaemia, non-Hodgkin’s lymphoma, and neuroblastoma, are commonly treated with these substances. Because certain alkaloids.	Alkaloids can cause harm to the liver, kidneys, and neurons; the main problem is toxicity. To address this restriction, well-conducted toxicity studies that focus on the specific mechanisms of action and toxicity levels of alkaloids are required. Other factors include short half-life, poor oral bioavailability, low solubility, and weak stability.	[22,23]
**Saponins**	Saponins are glycosides consisting of nonpolar triterpenes or sterols joined to hydrophilic oligosaccharide groups. These compounds target the NF-κB, PI3K-Akt-mTOR, MAPK, Wnt-β-catenin, JAK-STAT3, APMK, p53, and EGFR signalling pathways to produce their anticancer effects.	Its limited bioavailability, due to low permeability and poor water solubility, limits its clinical use.	[24,25]

**Table 2:** Green-synthesised NPs derived from herbal sources evaluated for PC therapy.

Nanoparticles	Biological Source	Size	Model	Advantages	Reference
**AgNPs**	*Azadirachta indica* leaf extract	~10–50 nm	PANC-1 cell lines	Induces apoptosis increase ROS, mitochondrial dysfunction, anti-proliferative, potential anti-metastatic effects	[104]
**AuNPs**	*Euterpe oleracea* (acai berry) and *Sambucus nigra* extracts	~20–40 nm	PANC-1 cell lines	Enhanced cytotoxicity vs. extracts alone, improved cellular uptake, and anti-proliferative effect	[105]
**Copper oxide nanoparticles (Cu_2_O NPs)**	*Sargassum latifolium* algal extract	40–50 nm (spherical-cubic)	PANC-1 cells	Apoptosis induction, S-phase cell cycle arrest, comparable to Arsenic trioxide	[104]
**Zinc oxide nanoparticles (ZnO NPs)**	*Sargassum muticum* seaweed extract with hyaluronan	Variable sizes	PANC-1 cells	Selective toxicity to cancer cells, G2/M cell cycle arrest	[106]
**Curcumin-loaded magnetic nanoparticles (MNP-CUR)**	*Curcuma longa* (turmeric) extract	10.5 Â ± 0.54 nm	HPAF-II, Panc-1 cells, xenograft mice	Enhanced bioavailability (2.5-fold), tumor targeting, and biocompatibility	[107]
**Selenium nanoparticles (SeNPs)**	*Gossypium barbadense* L. (cotton) extract	25–40 nm (estimated)	PANC1 cells	Downregulation of cancer cells, biocompatible synthesis	[108]
**Magnesium oxide nanoparticles (MgO NPs)**	*Momordica charantia* (bitter gourd) fruit extract	Not specified (green synthesis method)	PANC-1 cells,	Dose-dependent cytotoxicity, excellent hemocompatibility, enhanced bioavailability	[106]
**SeNPs—polysaccharide stabilized**	*Prunella vulgaris Linn*. clusters extract—heteroxylan PVP3-1	≈60 nm (spherical, uniform)	PC cells, mTOR signalling pathway inhibition	Autophagy and apoptosis induction via mTOR pathway inhibition, high stability	[109,110]
**Iron oxide nanoparticles (Fe_2_O_3_ NPs)**	*Aerva lanata* leaf extract	Variable sizes with excellent biocompatibility	PC cells, theranostic applications	Discriminatory toxicity in cancer cells, theranostic potential, antibiofilm activity	[111]
**AuNPs—Halodule uninervis extract**	*Halodule uninervis* seagrass ethanolic extract	Variable sizes with excellent stability	Capan-2 cells	Concentration and time-dependent cytotoxicity, apoptosis induction, and membrane blebbing	[112]
**SeNPs—Aspergillus flavus biosynthesis**	*Aspergillus flavus* fungal biosynthesis (green method)	28–78 nm (spherical shape)	PANC1 cells	Their chemoresistance and aggressive growth behaviour make them an appropriate in vitro model for assessing the anticancer potential of new treatment drugs.	[113]

**Table 3:** Recent Primary Studies of Herb-Derived Phytochemicals Delivered by Nanoparticles in Pancreatic Cancer and Related Models.

Delivery System	Particle Size (nm)	Herb/Phytochemical Source	Experimental Model & Main Outcomes	Reference
Aloe vera-derived extracellular vesicle-like particles with ursolic acid (EV-U) and Extracellular Vesicles loaded with Paclitaxel (EV-P)	179.3–227.1	*Aloe vera*	In vitro: PANC-1—inhibited migration & invasion; induced pyroptosis via ROS-GSDMD/E. In vivo: PANC-1 xenograft—tumour volume & decreased weight; no toxicity.	[119]
Curcumin-loaded emulsome nanoparticles (CurEm)	Not reported	Curcumin (*Curcuma longa*)	In vitro: PANC-1—apoptosis increased, G2/M cell-cycle arrest, migration and colony formation reduced; p53 and caspase activation compared with free curcumin.	[120]
Curcumin-loaded PLGA nanoparticles	~142	Curcumin (*Curcuma longa*)	In vitro: PANC-1—~3.5× lower IC_50_ vs. free curcumin; enhanced apoptosis & G2/M arrest; sustained release.	[121]
Dual-targeted protein-coated PLGA NPs (Esculetin + Curcumin)	Not reported	Esculetin (*Aesculus hippocastanum*) + Curcumin	In vitro: MIA PaCa-2—cell viability ↓; synergistic anti-proliferative effects; improved intracellular delivery & controlled release.	[122]
Berberine-loaded glyceryl monooleate nanoparticles	~128	Berberine (*Coptis chinensis*)	In vitro: PANC-1—enhanced apoptosis, migration inhibition, and AKT pathway suppression vs. free berberine.	[123]
Lawsone-encapsulated chitosan-folic acid PLGA nanoparticles	~173	Lawsone (*Lawsonia inermis*)	In vitro: PANC-1—induced apoptosis via BAX ↑/BCL-2 ↓; improved uptake via folate targeting.	[124]
EGCG-gold nanoparticle conjugates	~35	Epigallocatechin-3-gallate (*Camellia sinensis*)	In vitro: BxPC-3—increased ROS-mediated apoptosis and cytotoxicity vs. free EGCG.	[125]
Transferosome co-delivery of EGCG + Curcumin	~162	EGCG (Green tea) + Curcumin (*Curcuma longa*)	In vitro: PANC-1—apoptosis & uptake ↑; In vivo: PC xenograft—tumor growth suppression & improved bioavailability.	[126]

## Data Availability

No new data were created or analyzed in this study.

## Abbreviations

PDAC: pancreatic ductal adenocarcinoma; NP: nanoparticle; PC: pancreatic cancer; TME: tumor microenvironment; EPR: enhanced permeability and retention; CAFs: cancer-associated fibroblasts; ROS: reactive oxygen species; MMPs: matrix metalloproteinases; SLNs: solid lipid nanoparticles; NLCs: nanostructured lipid carriers; MSNs: mesoporous silica nanoparticles; ADAM9: a disintegrin and metalloproteinase 9; PEG: polyethylene glycol; CNTs: carbon nanotubes; SWCNTs: Single-walled carbon nanotubes; MWCNTs: multi-walled carbon nanotubes; GO: graphene oxide; rGO: reduced graphene oxide; GQDs: graphene quantum dots; CQDs: carbon quantum dots; NDs: nanodiamonds; SPIONs: superparamagnetic iron oxide nanoparticles; uIONPs: ultra-small iron oxide nanoparticles; PTT: photothermal therapy; PDT: photodynamic therapy; NIR: near-infrared; DOX: doxorubicin; ADR: adverse drug reaction; HDIs: herb–drug interactions; IFP: interstitial fluid pressure; silver nanoparticles: AgNPs; gold nanoparticles: AuNPs; ER: endoplasmic reticulum; TF: tissue factor; MNP-CUR: curcumin loaded magnetic nanoparticles; MgONPs: magnesium oxide nanoparticles; GMP: good manufacturing practice; ZnONPs: zinc oxide nanoparticles; Cu_2_ONPs: copper oxide nanoparticles; PTX: paclitaxel; PDX: patient-derived xenograft; XRD: x-ray diffraction.
